# Supplementary material for: Brain sex-dependent alterations after prolonged high fat diet exposure in mice
Source: Commun Biol. 2022 Nov 21;5:1276. doi: 10.1038/s42003-022-04214-x (PMC9681749; doi:10.1038/s42003-022-04214-x)
Supplement: Supplementary file 2 — Supplementary Material [file 42003_2022_4214_MOESM2_ESM.pdf]

Supplementary Information (SI)

Brain sex-dependent alterations in anterior cortex after prolonged high fat diet exposure:

Valentina Murtaj*[1,3] $, Silvia Penati*[5,6] &, Sara Belloli[2,3], Maria Foti[4], Angela Coliva[3], Angela Papagna[4], Cecilia Gotti[7], Elisa Toninelli[3], Remy Chiaffarelli[3.4]#, Stefano Mantero[8]£, Susanna Pucci[7], Michela Matteoli[5,6], Maria Luisa Malosio§[5,6], Rosa Maria Moresco§[2,3,4]

[1] PhD Program in Neuroscience, University of Milano-Bicocca, Monza (MB), Italy; murtaj.valentina@hsr.it (V.M.)

[2] Institute of Molecular Bioimaging and Physiology, CNR, 20090 Segrate (MI), Italy; sara.belloli@ibfm.cnr.it (S.B.); rosa.moresco@unimib.it (R.M.M.)

[3] Department of Nuclear Medicine, IRCCS San Raffaele Scientific Institute, 20132 Milan, Italy; murtaj.valentina@hsr.it (M.V.); coliva.angela@hsr.it (C.A.); toninelli.elisa@hsr.it (T.E.); remy.chiaffarelli@med.uni-tuebingen.de (R.C.)

4 Department of Medicine and Surgery, University of Milano-Bicocca, 20900 Monza, Italy; maria.foti@unimib.it (M.F.); angela.papagna@unimib.it (P.A.).

5 Institute of Neuroscience, National Research Council of Italy (CNR) c/o Humanitas Mirasole S.p.A, Via Manzoni 56, 20089 Rozzano (MI), Italy; penati.silvia11@gmail.com (S.P.); marialuisa.malosio@in.cnr.it (MLM); michela.matteoli@hunimed.eu (M.M.)

6 Laboratory of Pharmacology and Brain Pathology, Neuro Center, IRCCS Humanitas Research Hospital, Via Manzoni 56, 20089 Rozzano (MI), Italy penati.silvia11@gmail.com (S.P.); marialuisa.malosio@in.cnr.it (MLM); michela.matteoli@hunimed.eu (M.M.)

7 Institute of Neuroscience, National Research Council of Italy (CNR) c/o Università di Milano-Bicocca, Via Follereau 3, 20854 Vedano al Lambro (MB); cecilia.gotti@in.cnr.it (C.G.); susanna.pucci@in.cnr.it (S.Pu.)

8 Institute for Genetic and Biomedical Research, National Research Council of Italy (CNR) c/o Humanitas Mirasole S.p.A, Via Manzoni 56, 20089 Rozzano (MI), Italy; stefano.mantero@irgb.cnr.it (SM)

*shared contribution

§ correspondence: rosa.moresco@unimib.it, phone: 390226433817; marialuisa.malosio@in.cnr.it phone: +390282245150

$ Valentina Murtaj's current address: Neuroimmunology Unit, Institute of Experimental Neurology, IRCCS San Raffaele Hospital and Università San Raffaele Vita-Salute via Olgettina 32, 20132 Milano (Italy)

& Silvia Penati's current address: Department of Pathology and Immunology, Washington Univerisity School of Medicine, St. Louis, MO 63110, USA.

Remy Chiaffarelli's current address: Werner Siemens Imaging Center, Department of Preclinical Imaging and Radiopharmacy, Eberhard Karls Universität Tübingen, 72076 Tübingen (Germany)

£ Stefano Mantero's current address: DCSR- CNR, Via A. Corti 12, 20133 Milano (Italy)

Unimib, IBFM CNR: VM, SB, AC, RC; IN CNR, Humanitas: SPenati, MLM, MM; IN CNR: CG, SPucci; IRGB CNR: SM

Table of Contents

Supplementary Fig. 1……………………………………………………………………3
Supplementary Fig. 2……………………………………………………………………4
Supplementary Fig. 3…………………………………………………………………....5
Supplementary Fig. 4……………………………………………………………………6
Supplementary Fig. 5……………………………………………………………………7
Supplementary Fig. 6……………………………………………………………………8
Supplementary Fig. 7……………………………………………………………………9
Supplementary Fig. 8…………………………………………………………………..10
Supplementary Table 1…………………………………………………………………11
Supplementary Table 2…………………………………………………………………11
Supplementary Table 3…………………………………………………………………12
Supplementary Table 4…………………………………………………………………13

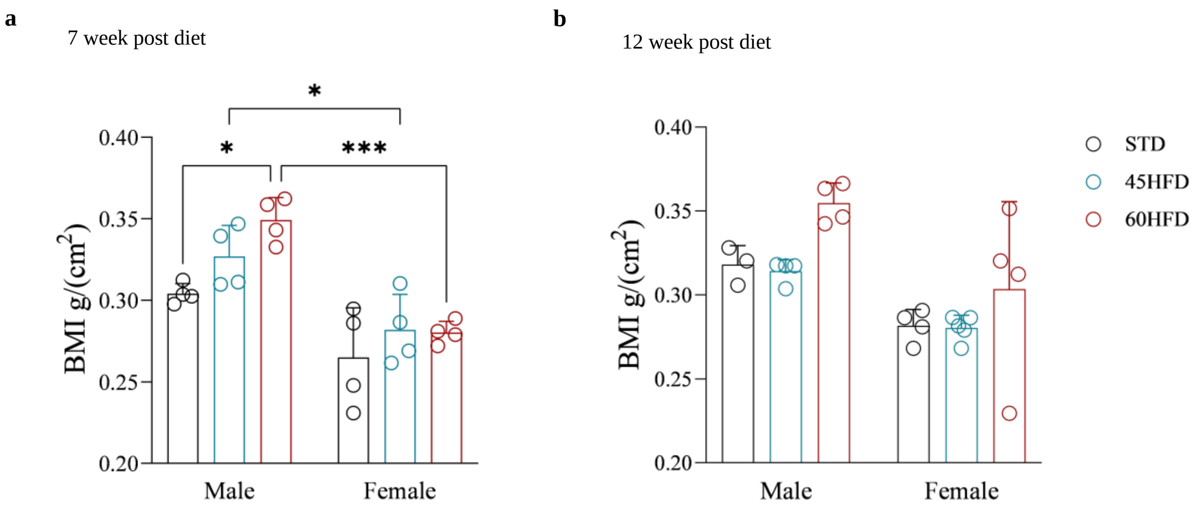

Supplementary Figure 1: BMI of male and female mice after 7 and 12 weeks of high fat diet consumption

(a) BMI in males and females at 7 weeks of diet. (b) BMI in males and females at 12 weeks of diet. Data were analyzed using Two-Way ANOVA including sex and diet as variable and were expressed as mean ±SD. * $p \leq 0.05$; *** $p \leq 0.001$

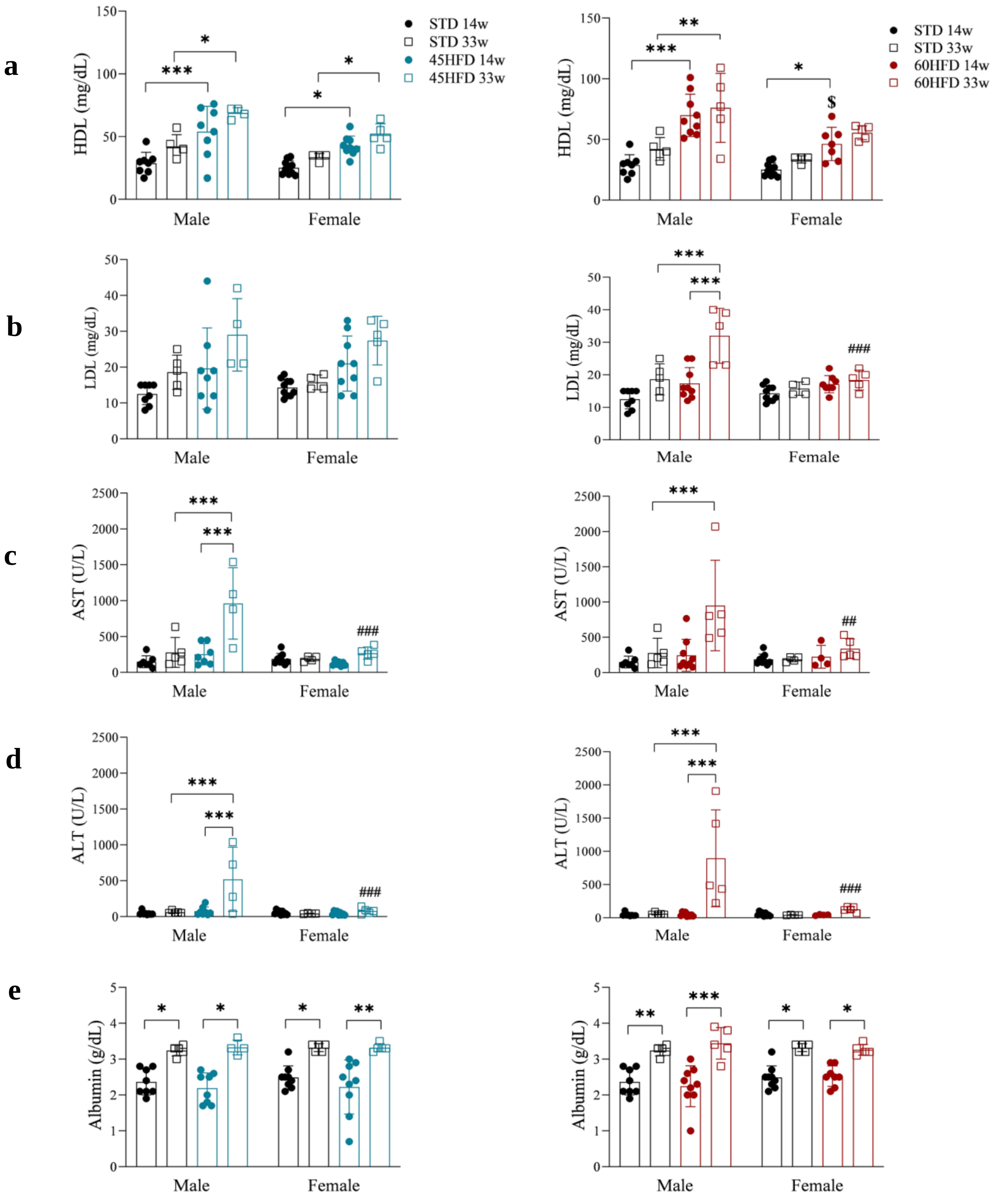

Supplementary Figure 2: Biochemical parameters of females and males fed with high fat diets for 14 and 33 weeks

45HFD (left panel) and 60HFD (right panel) vs STD male and female at 14 (14w) and 33 weeks (33w); **(a)** High Dense Lipoprotein (HDL); **(b)** Low Dense Lipoproteins (LDL); **(c)** Aspartate transaminase enzyme (AST); **(d)** Alanine transaminase enzyme (ALT); **(e)** Albumin (Alb). Data were analyzed using Three-Way ANOVA followed by Tukey's post hoc test for multiple comparison using sex and diet as covariate and are expressed as mean ± SD.

$ significance between HFD males and HFD females at 14 weeks; # significance between HFD males and HFD females at 33 weeks. * $p \leq 0.05$; *** $p \leq 0.001$

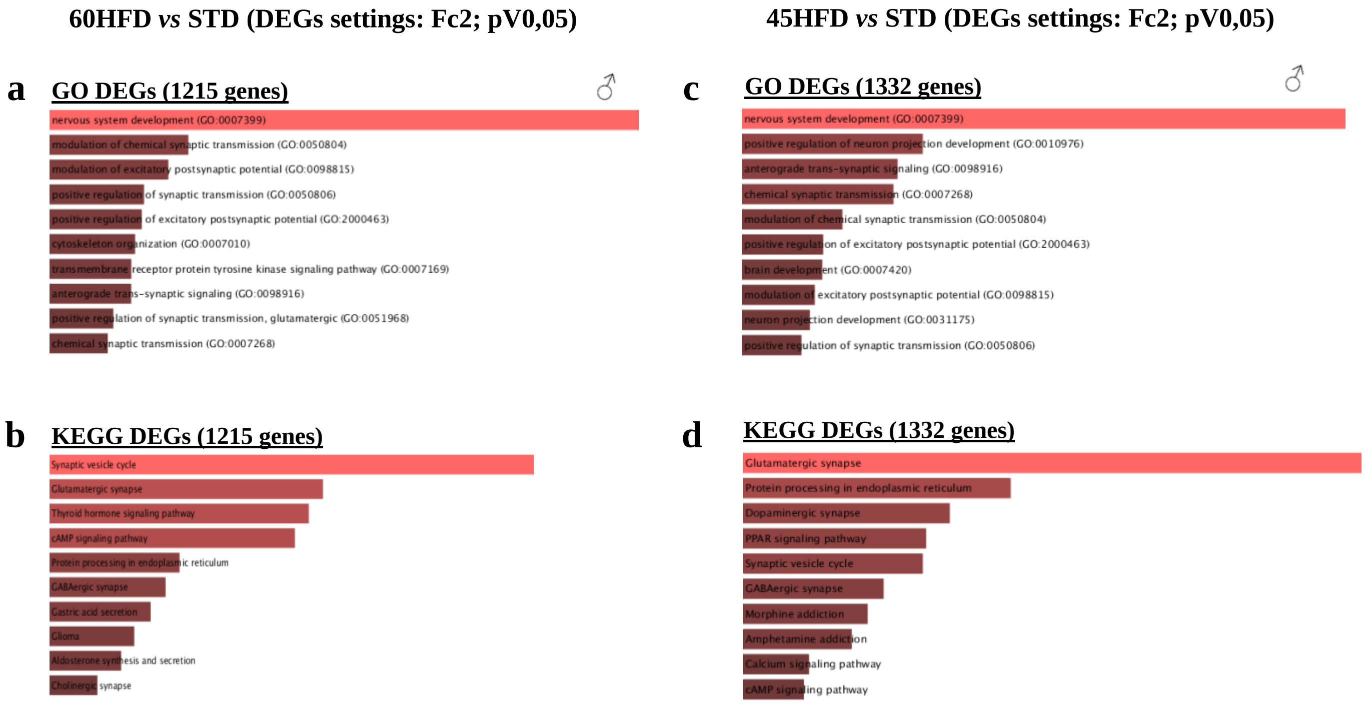

Supplementary Figure 3: **GO and KEGG analysis in 60HFD and 45HFD male mice**
(a) Differentially expressed genes (DEGs) derived from GO analysis in 60HFD male anterior cortex compared to STD control diet. **(b)** DEGs derived from KEGG analysis in 60HFD male anterior cortex compared to STD control diet. **(c)** Differentially expressed genes (DEGs) derived from GO analysis in 45HFD male anterior cortex compared to STD control diet. **(d)** DEGs derived from KEGG analysis in 45HFD male anterior cortex compared to STD control diet. Data were analyzed using One-Way ANOVA with the cutoff of p-value≤0.05 and fold change≥2. The length of the bar represents the significance of that specific gene-set or term. Significance of the specific gene-set is represented by the brightness of the color.

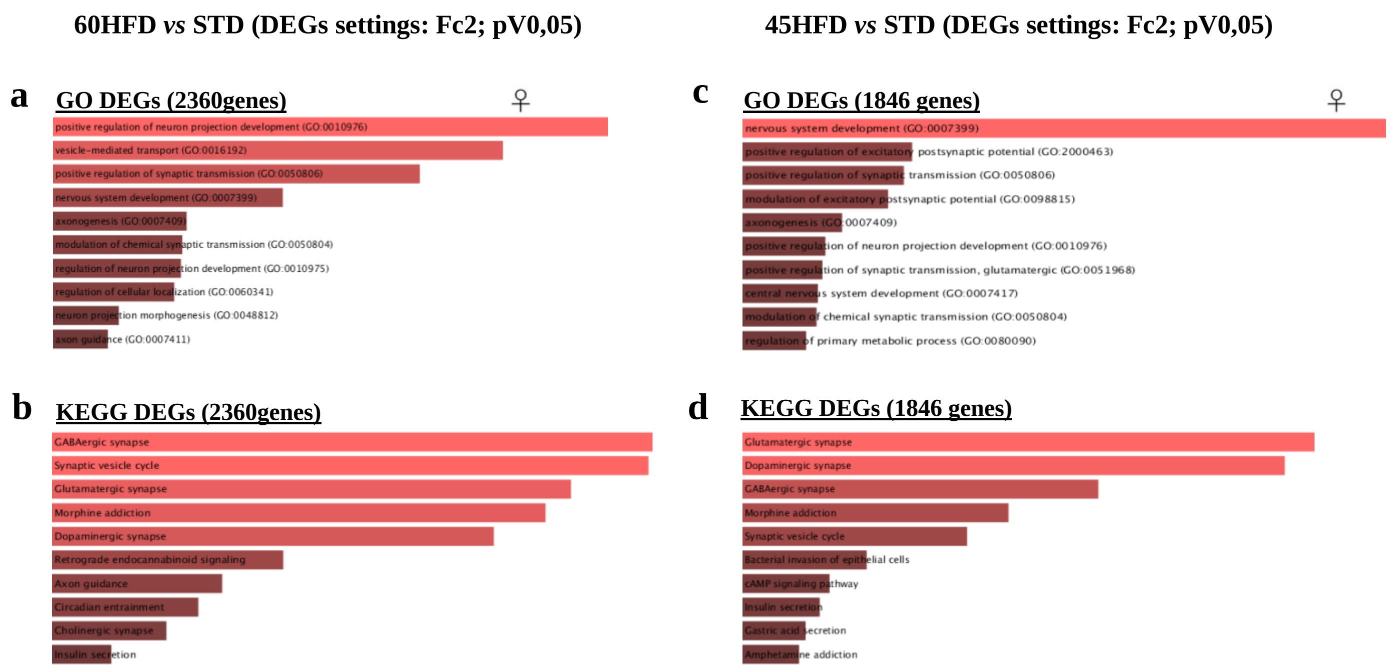

Supplementary Figure 4: **GO and KEGG analysis in 45HFD female mice**
(a) Differentially expressed genes (DEGs) derived from GO analysis in 60HFD female anterior cortex compared to STD control diet. **(b)** DEGs derived from KEGG analysis in 60HFD female anterior cortex compared to STD control diet. **(c)** Differentially expressed genes (DEGs) derived from GO analysis in 45HFD female anterior cortex compared to STD control diet. **(d)** DEGs derived from KEGG analysis in 45HFD female anterior cortex compared to STD control diet. Data were analyzed using One-Way ANOVA with the cutoff of p-value≤0.05 and fold change≥2. The length of the bar represents the significance of that specific gene-set or term. Significance of the specific gene-set is represented by the brightness of the color

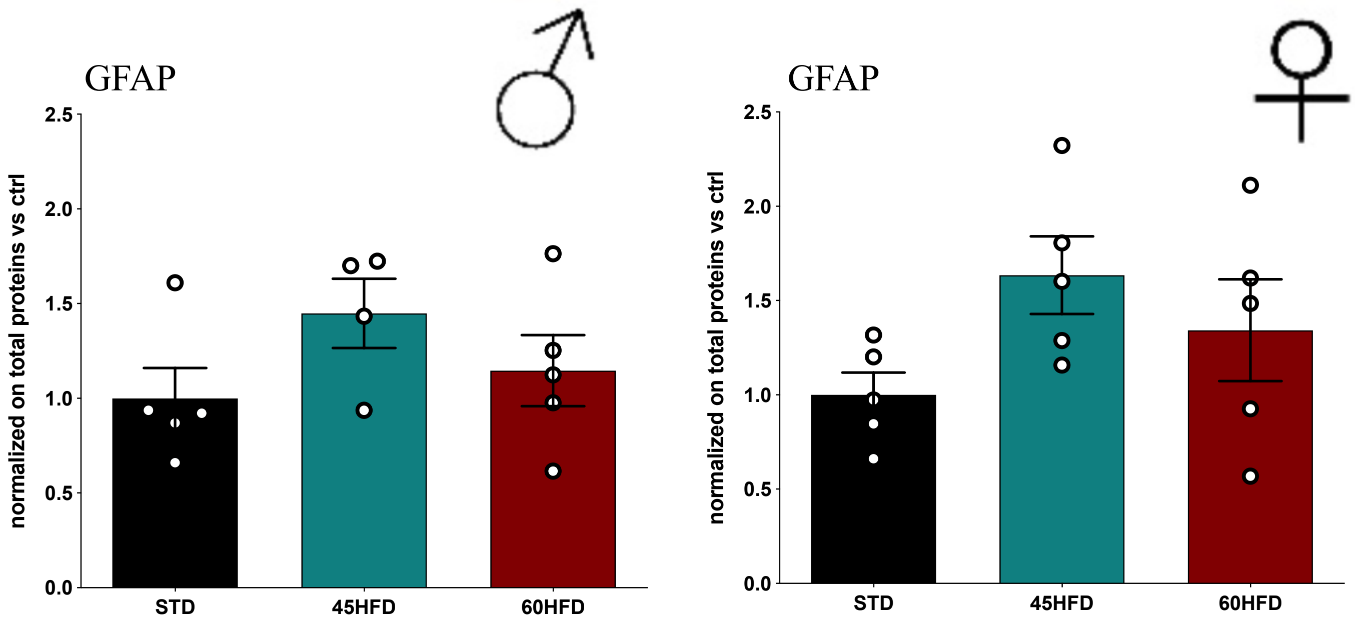

Supplementary Figure 5. Western blot quantification of Glial Fibrillary Acidic Protein (GFAP) protein in male and female anterior cortex at 35 weeks of diet.
Data obtained from technical replicate analyses, normalized on total proteins, were analyzed using Kruskal–Wallis test followed by Dunn's post hoc test and are expressed as mean ± S.E.M. vs STD

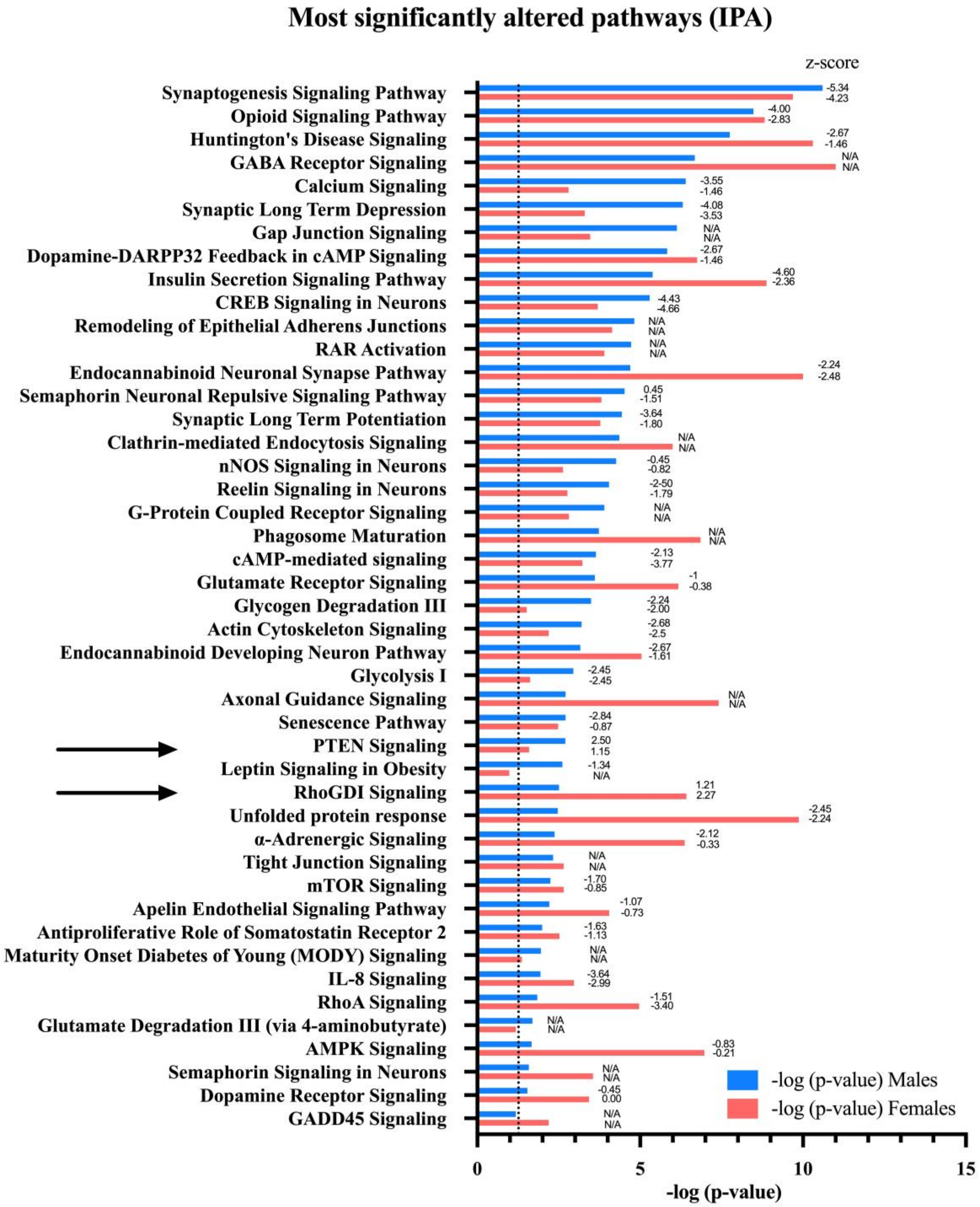

Supplementary Figure 6: Ingenuity Pathway Analysis (IPA) results in Anterior Cortex. The most significantly altered pathways common to males (blue bars) and females (red bars) in HFD60 vs CTRL in the canonical pathway analysis are presented from the more to the less significant. X-axis: –log (p-value). The dotted line represents the value of 1.3, corresponding to a p-value of 0.05. IPA's z-scores are indicated at the end of each bar. N/A means that IPA is not able to assign an activity pattern to a given pathway. Arrows on the left indicate the pathways, which had a positive z-score**.**

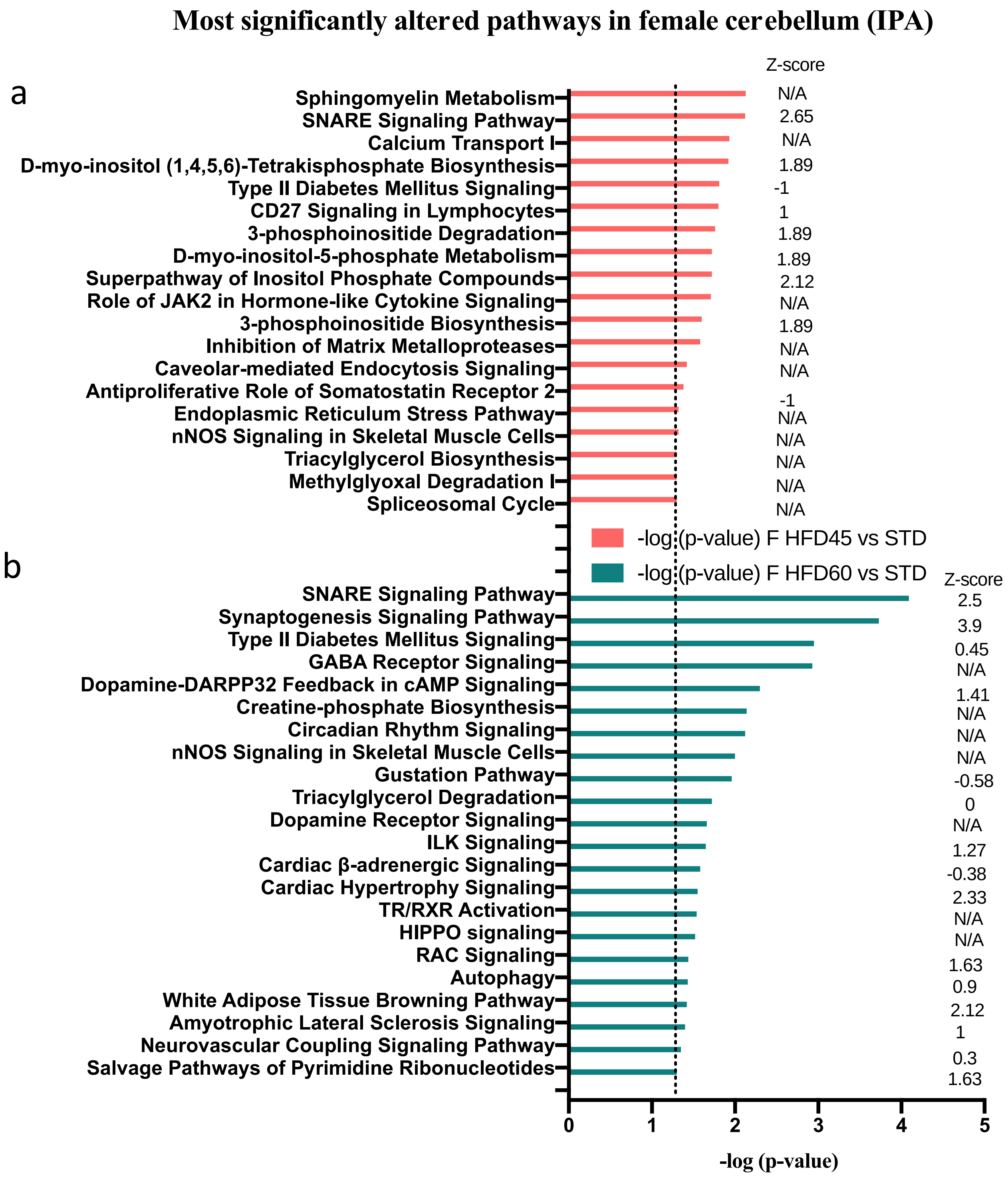

Supplementary Figure 7: Ingenuity Pathway Analysis (IPA) results in cerebellum. The most significantly altered pathways in female cerebellum in HFD45 vs STD (a) and in HFD60 vs STD (b) in the canonical pathway analysis are presented from the more to the less significant. X-axis: -log (p-value). The dotted line corresponds to 1.3 (-log 0.05). IPA's z-scores are indicated at the end of each bar. N/A: IPA is not able to assign an activity pattern to a pathway.

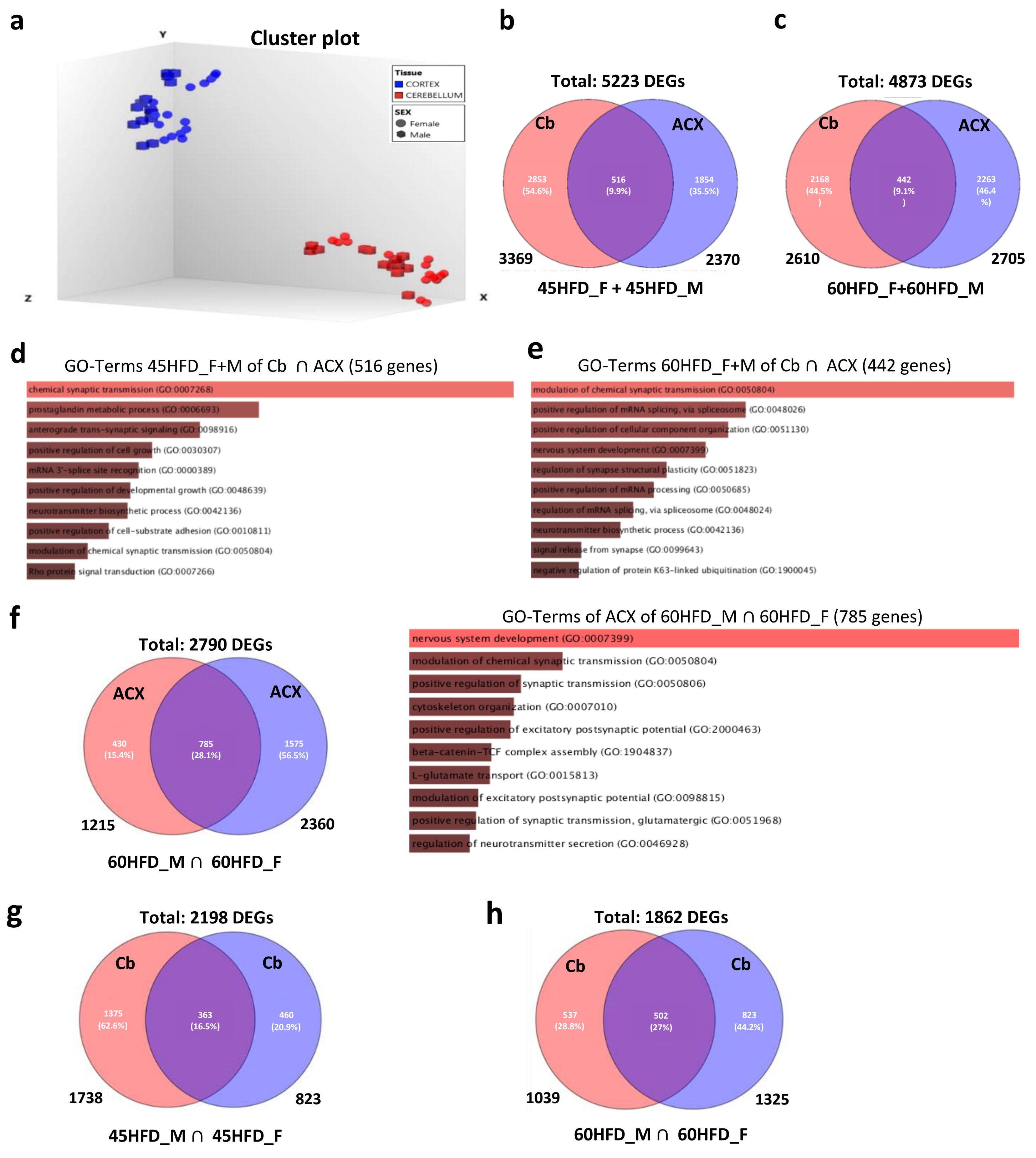

Supplementary Figure 8. Venn diagram analyses
a. Principal Component analysis of males and females DEGs in Cb and ACX. DEGs in Cb and ACX show a high degree of segregation indicating specific gene expression patterns in the two brain regions. b-c Differentially Expressed Genes (DEGs) common to Cb and ACX in animals fed on 45HFD (516 (9.9%), b) and 60HFD (442 (9.1%), c). d-e GO-Terms significantly enriched in DEGs common to Cb and ACX of Males and Females fed on 45HFD (516 (9.9%, d) and of Males and Females fed on 60HFD (442 (9.1%); e. f. Venn diagram representation of DEGs of the ACX common to males and females fed on 60HFD (785 (28.1%) and GO-Terms enrichment. g-h Venn diagram representation of DEGs of the Cb ACX common to males and females fed on 45HFD (g) and on 60HFD (h). The GO bars are sorted by p-Value rankings represented by the bar length and colors indicating significant enrichments below the threshold of 0.05.

Supplementary TABLE 1 High Fat Diet and Standard Diet Fat composition

FAT	%	Saturated	Monounsaturated	Polyunsaturated	Main ingredient
**STD**	10%	23%	30%	47%	WHEAT
**45HFD**	45%	31%	36%	33%	LARD
**60HFD**	60%	32%	36%	32%	LARD

Supplementary TABLE 2 Sample size for each experimental task

	**Task**	**BMI**		**PET §**						**GTT**	**Post mortem***	**Hemato#**	
				FDG PET			VC701 PET						
	**Time (weeks)**	14	33	7	12	31	7	12	31	31	33	14	33
**M**	**60% HFD**	5	5	8	8	3	6	6	3	5	5	10	5
	**45% HFD**	5	5	8	8	4	6	6	3	5	4	10	5
	**STD**	5	5	8	7	4	6	6	3	5	5	10	5
**F**	**60% HFD**	5	5	8	8	4	6	6	3	5	5	10	5
	**45% HFD**	5	5	8	8	4	6	6	3	5	5	10	5
	**STD**	5	5	8	8	4	6	6	3	5	5	10	5

§ PET studies with FDG and VC701 were performed starting during week 7, 12 and 31 on different days with a minimum distance of 48 hours. For each PET section, a maximum of 12 mice for FDG and 9 for VC701 were acquired with the same radiopharmaceutical preparation. Male and Female started the diet and were processed in separate days. #Hematochemistry 14 w: 5 mice from PET group (group 1) and liver group (group 2) were sacrificied and used also for hematochemistry analysis. * Female STD group for western blot, n=4; 1 Male of 45HFD, n=4: spontaneously died.

Supplementary TABLE 3: Spearman r and p values of PET [^{18}F]FDG correlations analysis for males (A) and females (B)

A	**Glycaema**		**BMI**		**Tot. Chol**		**HDL**		**LDL**		**AST**		**ALT**		**Albumin**	
31 weeks - Males	r	p	r	p	r	p	r	p	r	p	r	p	r	p	r	p
**Olfactory bulb**	0,47	0,179	**0,71**	**0,027**	**0,79**	**0,009**	0,69	0,033	**0,81**	**0,006**	0,54	0,114	0,52	0,133	-0,13	0,744
**Anterior cortex**	0,37	0,296	0,52	0,133	0,66	0,044	0,55	0,101	**0,81**	**0,006**	0,48	0,166	0,43	0,218	0,07	0,869
**Whole cortex**	0,15	0,674	0,45	0,192	0,66	0,043	0,62	0,061	0,69	0,031	0,39	0,264	0,38	0,272	-0,11	0,764
**Striatum**	0,33	0,353	0,38	0,282	0,61	0,067	0,51	0,134	0,69	0,030	0,42	0,227	0,38	0,282	0,22	0,546
**Hippocampus**	-0,22	0,542	-0,01	0,980	0,22	0,542	0,18	0,609	0,17	0,630	-0,16	0,649	-0,01	0,980	-0,08	0,829
**Hypthalamus**	-0,01	0,994	0,08	0,831	0,29	0,420	0,19	0,602	0,37	0,296	-0,02	0,952	-0,01	0,979	0,19	0,608
**Thalamus**	-0,22	0,542	-0,27	0,453	-0,15	0,688	-0,17	0,639	-0,17	0,635	-0,18	0,624	0,04	0,926	0,16	0,668
**Brainstem**	-0,33	0,349	-0,31	0,388	-0,40	0,254	-0,39	0,263	-0,48	0,163	-0,36	0,303	-0,13	0,712	-0,21	0,560
**Cerebellum**	-0,51	0,139	**-0,72**	**0,024**	**-0,89**	**0,001**	**-0,89**	**0,001**	-0,55	0,105	-0,61	0,069	-0,46	0,186	0,23	0,536
**SUV whole brain**	**-0,71**	**0,027**	-0,54	0,114	-0,62	0,060	-0,57	0,093	**-0,71**	**0,024**	**-0,76**	**0,015**	**-0,72**	**0,023**	0,10	0,800

**B**	**Glycaema**		**BMI**		**Tot. Chol**		**HDL**		**LDL**		**AST**		**ALT**		**Albumin**	
31 weeks - Females	**r**	**p**	**r**	**p**	**r**	**p**	**r**	**p**	**r**	**p**	**r**	**p**	**r**	**p**	**r**	**p**
**Olfactory bulb**	0,39	0,257	0,68	0,025	0,25	0,446	0,43	0,184	-0,19	0,578	0,38	0,251	**0,73**	**0,013**	0,01	0,982
**Anterior cortex**	0,59	0,077	0,10	0,760	0,31	0,350	0,21	0,543	-0,01	0,973	0,14	0,694	0,46	0,155	0,17	0,607
**Whole cortex**	0,13	0,727	-0,05	0,895	0,12	0,715	0,23	0,495	0,22	0,516	0,19	0,581	0,41	0,216	0,17	0,606
**Striatum**	0,28	0,431	0,34	0,297	0,42	0,197	0,56	0,075	-0,04	0,900	**0,74**	**0,011**	0,62	0,048	0,34	0,304
**Hippocampus**	0,09	0,803	-0,02	0,949	0,04	0,909	0,34	0,308	-0,30	0,366	0,28	0,400	0,09	0,799	0,05	0,900
**Hypthalamus**	0,17	0,631	0,53	0,099	0,13	0,699	0,31	0,351	-0,24	0,472	0,43	0,188	0,21	0,533	0,10	0,772
**Thalamus**	0,13	0,720	0,23	0,498	0,24	0,466	0,49	0,132	-0,22	0,503	0,64	0,040	0,51	0,114	0,15	0,655
**Brainstem**	0,06	0,872	0,44	0,177	-0,03	0,921	0,02	0,953	-0,43	0,189	-0,03	0,940	-0,11	0,739	-0,21	0,530
**Cerebellum**	-0,41	0,235	-0,54	0,090	-0,27	0,417	-0,46	0,158	-0,02	0,964	-0,59	0,061	**-0,70**	**0,021**	-0,09	0,776
**SUV whole brain**	-0,15	0,684	-0,03	0,930	0,19	0,570	0,28	0,405	0,40	0,221	0,36	0,273	0,47	0,146	-0,59	0,061

Supplementary TABLE 4 Spearman r and p values of PET [^{18}F]VC701 correlations analysis for males (A) and females (B)

A	**Glycaemia**		**BMI**		**Tot. Chol**		**HDL**		**LDL**		**AST**		**ALT**		**Albumin**	
31 weeks - Males	r	p	r	p	r	p	r	p	r	p	r	p	r	p	r	p
**Olfactory bulb**	0,43	0,299	0,47	0,213	0,55	0,171	0,24	0,582	**0,78**	**0,026**	0,64	0,096	0,71	0,058	0,76	0,071
**Anterior cortex**	0,38	0,360	0,60	0,097	**0,79**	**0,028**	0,40	0,327	**0,80**	**0,023**	0,62	0,115	**0,74**	**0,046**	0,76	0,071
**Whole cortex**	0,29	0,501	0,63	0,076	**0,81**	**0,022**	0,43	0,299	0,71	0,056	0,52	0,197	0,67	0,083	0,63	0,143
**Striatum**	0,29	0,501	0,57	0,121	**0,74**	**0,046**	0,33	0,428	**0,88**	**0,007**	0,50	0,216	0,62	0,115	0,76	0,071
**Hippocampus**	0,38	0,360	0,60	0,097	**0,79**	**0,028**	0,40	0,327	**0,80**	**0,023**	0,62	0,115	**0,74**	**0,046**	0,76	0,071
**Hypthalamus**	0,38	0,360	0,60	0,097	**0,79**	**0,028**	0,40	0,327	**0,80**	**0,023**	0,62	0,115	**0,74**	**0,046**	0,76	0,071
**Thalamus**	0,19	0,665	0,53	0,148	0,69	0,069	0,31	0,462	**0,83**	**0,014**	0,43	0,299	0,55	0,171	0,76	0,071
**Brainstem**	0,29	0,501	0,48	0,194	**0,74**	**0,046**	0,38	0,360	**0,75**	**0,040**	0,55	0,171	0,67	0,083	0,76	0,071
**Cerebellum**	0,48	0,243	0,67	0,059	**0,83**	**0,015**	0,45	0,268	0,63	0,106	0,67	0,083	**0,79**	**0,028**	0,76	0,071

B	**Glycaemia**		**BMI**		**Tot. Chol**		**HDL**		**LDL**		**AST**		**ALT**		**Albumin**	
31 weeks - Females	r	p	r	p	r	p	r	p	r	p	r	p	r	p	r	p
**Olfactory bulb**	0,48	0,243	0,08	0,854	0,05	0,912	0,00	>0,999	0,19	0,665	0,33	0,428	0,57	0,151	0,13	0,857
**Anterior cortex**	0,40	0,327	0,00	>0,999	-0,07	0,880	-0,24	0,582	0,19	0,665	0,07	0,882	0,45	0,268	-0,13	0,857
**Whole cortex**	0,41	0,314	0,01	0,987	0,02	0,973	-0,05	0,916	0,24	0,564	0,26	0,523	0,54	0,177	0,06	0,929
**Striatum**	0,44	0,273	0,03	0,942	-0,06	0,886	-0,19	0,647	0,14	0,736	0,14	0,736	0,49	0,222	-0,06	0,929
**Hippocampus**	0,45	0,268	0,08	0,854	-0,05	0,912	-0,12	0,793	0,02	0,977	0,29	0,501	0,50	0,216	0,00	>0,999
**Hypthalamus**	0,44	0,273	0,04	0,924	0,03	0,957	0,00	>0,999	0,19	0,665	0,33	0,428	0,57	0,151	0,13	0,857
**Thalamus**	0,40	0,327	-0,02	0,973	-0,10	0,810	-0,14	0,752	0,10	0,840	0,21	0,619	0,52	0,197	0,00	>0,999
**Brainstem**	0,45	0,268	0,08	0,854	-0,05	0,912	-0,12	0,793	0,02	0,977	0,29	0,501	0,50	0,216	0,00	>0,999
**Cerebellum**	0,52	0,197	0,15	0,700	0,00	>0,999	-0,12	0,793	0,02	0,977	0,29	0,501	0,50	0,216	0,00	>0,999
