# Supplementary material for: Brain sex-dependent alterations after prolonged high fat diet exposure in mice
Source: Commun Biol. 2022 Nov 21;5:1276. doi: 10.1038/s42003-022-04214-x (PMC9681749; doi:10.1038/s42003-022-04214-x)
Supplement: Supplementary file 3 — Description of Additional Supplementary Files [file 42003_2022_4214_MOESM3_ESM.pdf]

Description of Additional Supplementary Files

File name: Supplementary Data 1
Description: The source data behind Figures presented in main test and supplementary.

File name: Supplementary Data 2
Description: common genes between cerebellum and anterior cortex in females and in males differentially expressed in 45HFD or 60HFD and STD.
