# Supplementary material for: Brain sex-dependent alterations after prolonged high fat diet exposure in mice
Source: Commun Biol. 2022 Nov 21;5:1276. doi: 10.1038/s42003-022-04214-x (PMC9681749; doi:10.1038/s42003-022-04214-x)
Supplement: Supplementary file 6 — Reporting Summary [file 42003_2022_4214_MOESM6_ESM.pdf]

nature research

Corresponding author(s): Rosa Maria Moresco & Maria Luisa Malosio

Last updated by author(s): 10/17/2022

Reporting Summary

Nature Research wishes to improve the reproducibility of the work that we publish. This form provides structure for consistency and transparency in reporting. For further information on Nature Research policies, see our Editorial Policies and the Editorial Policy Checklist.

Statistics

For all statistical analyses, confirm that the following items are present in the figure legend, table legend, main text, or Methods section.

n/a	Confirmed	
☐	☒	The exact sample size (n) for each experimental group/condition, given as a discrete number and unit of measurement
☐	☒	A statement on whether measurements were taken from distinct samples or whether the same sample was measured repeatedly
☐	☒	The statistical test(s) used AND whether they are one- or two-sided<br>*Only common tests should be described solely by name; describe more complex techniques in the Methods section.*
☒	☐	A description of all covariates tested
☐	☒	A description of any assumptions or corrections, such as tests of normality and adjustment for multiple comparisons
☐	☒	A full description of the statistical parameters including central tendency (e.g. means) or other basic estimates (e.g. regression coefficient) AND variation (e.g. standard deviation) or associated estimates of uncertainty (e.g. confidence intervals)
☐	☒	For null hypothesis testing, the test statistic (e.g. F, t, r) with confidence intervals, effect sizes, degrees of freedom and P value noted<br>*Give P values as exact values whenever suitable.*
☒	☐	For Bayesian analysis, information on the choice of priors and Markov chain Monte Carlo settings
☒	☐	For hierarchical and complex designs, identification of the appropriate level for tests and full reporting of outcomes
☒	☐	Estimates of effect sizes (e.g. Cohen's d, Pearson's r), indicating how they were calculated

Our web collection on statistics for biologists contains articles on many of the points above.

Software and code

Policy information about availability of computer code

Data collection	*Provide a description of all commercial, open source and custom code used to collect the data in this study, specifying the version used OR state that no software was used.*
Data analysis	*Provide a description of all commercial, open source and custom code used to analyse the data in this study, specifying the version used OR state that no software was used.*

For manuscripts utilizing custom algorithms or software that are central to the research but not yet described in published literature, software must be made available to editors and reviewers. We strongly encourage code deposition in a community repository (e.g. GitHub). See the Nature Research guidelines for submitting code & software for further information.

Data

Policy information about availability of data

All manuscripts must include a data availability statement. This statement should provide the following information, where applicable:

- Accession codes, unique identifiers, or web links for publicly available datasets
- A list of figures that have associated raw data
- A description of any restrictions on data availability

The datasets generated during the current study supporting the findings of this study are available from the corresponding authors upon reasonable request. The raw data are not yet publicly available.

Field-specific reporting

Please select the one below that is the best fit for your research. If you are not sure, read the appropriate sections before making your selection.

☒ Life sciences ☐ Behavioural & social sciences ☐ Ecological, evolutionary & environmental sciences

For a reference copy of the document with all sections, see nature.com/documents/nr-reporting-summary-flat.pdf

Life sciences study design

All studies must disclose on these points even when the disclosure is negative.

Sample size: The minimum sample size was calculated using a separate set of brain [18F]-FDG-PET data, using G*Power software and considering a power of 0.8, alpha of 0.05 and an estimated effect size calculated considering a diet induced relevant modification equal to 3 Standard Deviation of the mean value of uptake in the anterior cortex. Given these parameters, total sample size for three conditions was equal to 9.

Data exclusions: *Describe any data exclusions. If no data were excluded from the analyses, state so OR if data were excluded, describe the exclusions and the rationale behind them, indicating whether exclusion criteria were pre-established.*

Replication: For western blot analyses at least two technical replicates of all experiments were performed.

Randomization: randomization of body weight in the different diet groups was done at the beginning of the treatment

Blinding: No blinding was done

Reporting for specific materials, systems and methods

We require information from authors about some types of materials, experimental systems and methods used in many studies. Here, indicate whether each material, system or method listed is relevant to your study. If you are not sure if a list item applies to your research, read the appropriate section before selecting a response.

Materials & experimental systems

n/a	Involved in the study	
☐	☒	Antibodies
☒	☐	Eukaryotic cell lines
☒	☐	Palaeontology and archaeology
☐	☒	Animals and other organisms
☒	☐	Human research participants
☒	☐	Clinical data
☐	☐	Dual use research of concern

Methods

n/a	Involved in the study	
☒	☐	ChIP-seq
☒	☐	Flow cytometry
☒	☐	MRI-based neuroimaging

Antibodies

Antibodies used:
Anti-GluA1 and GluA2/3 AMPA receptors antibodies were developed by the co-author Cecilia Gotti (reference n. 70).
Anti-NR1 (NMDA Receptor Subunit 1) antibody was clone (clone 54.1) cat. N 556308 from BD Pharmingen, now sold by Merck cat. n. MAB363
Anti GluN2A (NMDA Receptor Subunit 2A) antibody was clone A3-2D10, Invitrogen cat n. 32-0600 sold by Life technologies/ Thermofisher
The anti-GluN2B (NMDA Receptor Subunit 2B) antibody was clone N59/20), produced by UC Davis/NIH NeuroMab Facility Antibodies Incorporated, Davis, CA, USA cat. Number?
anti-PSD95 (clone K28/43) produced by UC Davis/NIH NeuroMab Facility Antibodies Incorporated, Davis, CA, USA is commercialized by several companies (Merck, Biolegend, Abcam)
Anti-human AKT1 mouse IgG1, ID #610860 was purchased from BD Transduction Laboratories, Invitrogen
Anti-human pSer473-AKT1 rabbit IgG, ID #44-621G from Invitrogen was purchased from Thermo Fisher Scientific.
Anti GAPDH Ab (FL-335) sc 25778 rabbit polyclonal Ab produced and sold by Santa Cruz Biotechnology, INC.
Anti Iba-1 Ab is a rabbit polyclonal antibody cat n. 016.20001, sold by WAKO Fujifilm.

Validation: The specificity of the affinity-purified anti-GluA1 and GluA2/3 AMPA receptors was tested by Western blotting studies using cells transfected and non-transfected with GluA1, GluA2 and GluA3 cDNAs (see Pistillo F et al. ref n. 70 of the manuscript)Anti-NR1 (NMDA Receptor Subunit 1) clone 54.1 was raised against aa 660-811 of the rat protein, which have a high identity with the mouse protein and the antibody was validate for western blot, immunocytochemistry and immunofluorescence (published in Fernandes et al. PLOS ONE 2014 9(6) e99958).
The Anti GluN2A (NMDA Receptor Subunit 2A) antibody was clone A3-2D10, the antibody was raised against a fusion protein of the rat sequence, which is highly homologous to mouse, it was validated for ELISA, Immunoprecipitation, Immunostaining and western

blotting. The data sheet can be found at http://tools.thermofisher.com › 320600_Rev1008
The anti-GluN2B (NMDA Receptor Subunit 2B antibody was clone N59/20), produced by UC Davis/NIH NeuroMab Facility, was raised against a fusion protein of amino acids 20-271 (extracellular N-terminus) of rat GluN2B/NR2B subunit. The sequence is 99% identity with mouse sequence and the antibody was validated for immunoblotting, immunocytochemistry, immunohistochemistry and immunoprecipitation. See datasheet https://neuromab.ucdavis.edu/datasheet/N59_20.pdf
PSD95 clone K28/43 was raised against a fusion protein of amino acids 77-299 (PDZ domains 1 and 2) of human PSD-95. The sequence is 99% identity (221/223 amino acids identical) with mouse sequence and the antibody was validated for mmunoblot, Immunocytochemistry, Immunogold EM and Immunoprecipitation. See datahseet https://neuromab.ucdavis.edu › datasheet › K28_43. It has been cited at least by 70 publications https://www.citeab.com/antibodies/227407-mabn68-anti-psd95-antibody-clone-k28-43
Anti-human AKT1 mouse IgG1, ID #610860 was purchased from BD Transduction Laboratories, Invitrogen 40 citations have been found for this antibody at https://www.citeab.com/antibodies/2410293-610860-bd-transduction-laboratories-purified-mouse
Anti-human pSer473-AKT1 rabbit IgG, ID #44-621G from Invitrogen was purchased from Thermo Fisher Scientific. The website refers to numerous publications: https://www.thermofisher.com/antibody/product/Phospho-AKT1-Ser473-Antibody-clone-14-6-Monoclonal/44-621G
The rabbit GAPDH polyclonal Ab is raised against the aminoacid 1-335 of full length human GAPDH and cross-reacts with the mouse protein. https://www.scbt.com/it/p/gapdh-antibody-fl-335 has been cited by 621 publications.
Anti Iba-1 was raised against a synthetic C-terminal peptide of the protein, it is validated for western blot by the producer and cited in 131 publications https://labchem-wako.fujifilm.com/europe/product/detail/W01W0101-2000.html

Animals and other organisms

Policy information about studies involving animals; ARRIVE guidelines recommended for reporting animal research

Laboratory animals
Five weeks old male and female C57Bl/6J mice were used

Wild animals
Provide details on animals observed in or captured in the field; report species, sex and age where possible. Describe how animals were caught and transported and what happened to captive animals after the study (if killed, explain why and describe method; if released, say where and when) OR state that the study did not involve wild animals.

Field-collected samples
For laboratory work with field-collected samples, describe all relevant parameters such as housing, maintenance, temperature, photoperiod and end-of-experiment protocol OR state that the study did not involve samples collected from the field.

Ethics oversight
Identify the organization(s) that approved or provided guidance on the study protocol, OR state that no ethical approval or guidance was required and explain why not.

Note that full information on the approval of the study protocol must also be provided in the manuscript.

Dual use research of concern

Policy information about dual use research of concern

Hazards

Could the accidental, deliberate or reckless misuse of agents or technologies generated in the work, or the application of information presented in the manuscript, pose a threat to:

No	Yes	
☒	☐	Public health
☒	☐	National security
☒	☐	Crops and/or livestock
☒	☐	Ecosystems
☒	☐	Any other significant area

Experiments of concern

Does the work involve any of these experiments of concern:

No	Yes	
☒	☐	Demonstrate how to render a vaccine ineffective
☒	☐	Confer resistance to therapeutically useful antibiotics or antiviral agents
☒	☐	Enhance the virulence of a pathogen or render a nonpathogen virulent
☒	☐	Increase transmissibility of a pathogen
☒	☐	Alter the host range of a pathogen
☒	☐	Enable evasion of diagnostic/detection modalities
☒	☐	Enable the weaponization of a biological agent or toxin
☒	☐	Any other potentially harmful combination of experiments and agents
